# Fellow and Attending Surgeon Operative Notes are Deficient in Reporting Established Quality Indicators for Roux-en-Y Gastric Bypass: A Preliminary Retrospective Analysis of Operative Dictation

**DOI:** 10.7759/cureus.4535

**Published:** 2019-04-24

**Authors:** Ashley Vergis, Krista Hardy, Shannon Stogryn

**Affiliations:** 1 Surgery, University of Manitoba, Winnipeg, CAN

**Keywords:** operative dictation, roux y gastric bypass, trainee, attending, quality

## Abstract

Introduction

Surgeons must dictate the important components of any invasive procedure in a comprehensive, yet concise, operative report. This documentation is vital for communicating operative events and has implications for providing additional healthcare and planning future operations. The quality of surgical care may be impaired in the absence of such communication. Evidence suggests that the quality of reports dictated by trainees and surgeons is poor despite its importance. This investigation analyzed and compared the quality of fellow and staff surgeon Roux-en-Y Gastric Bypass (RYGB) narrative dictations against validated and reliable quality indicators (QIs) for this procedure.

Methods

A total of 40 bariatric fellow reports and 40 attending RYGB narrative reports were retrospectively analyzed.

Results

Fellows had a mean completion of 66.4% +/- 3.1% as compared to 61.5% +/- 7.6% for attendings (p<0.0001). Fellows statistically outperformed attendings on all subsections except patient, closure, and postoperative details. Attendings statistically outperformed fellows on closure details only (63.8 +/- 7.5 vs 50.5 +/- 12.0, p=0.002).

Conclusions

Bariatric surgery trainees outperform attending surgeons in RYGB operative dictation. The clinical significance of this difference is unknown. However, both groups are deficient in reporting at least one-third of items deemed essential to RYGB operative reporting. This indicates a need for further education in RYGB dictation for practicing surgeons and trainees. It also lends interest in exploring alternative forms of operative communication such as synoptic operative reporting in bariatric surgery.

## Introduction

Surgeons must record the findings and important components of an operative procedure in a succinct and thorough operative report. This documentation is the key format for communicating intraoperative events with health professionals and has far-reaching implications for planning future operative procedures and adjuvant care [1]. Operative reports also have an important role in quality assurance, research, billing, and medical-legal conflicts [2].

Currently, "standard" practice is for the responsible surgeon or delegate to generate a narrative report where the steps, rationale, and indications for the procedure are recorded. However, narrative reports have been scrutinized extensively regarding quality, particularly concerning incomplete or inaccurate documentation of important information [3-15]. In an early review of rectal cancer surgical reports, only 45.9% of items deemed important by a consensus panel could be retrieved from the dictations while less pertinent information could be retrieved up to 97% of the time [3]. This theme has permeated the literature in narrative reporting since [3-15].

Many surgeons and learners report minimal formal education in operative notation despite the ongoing scrutiny [16-17]. Furthermore, they believe the quality of reports dictated by both staff surgeons and trainees is mediocre [16]. This has been borne out extensively in the general operative reporting literature. In bariatric surgery, survey data indicates that those surgeons similarly feel the quality of bariatric operative reports are poor and surgical care may be impaired because of it [18].

However, the completeness of operative reporting in bariatric surgery has been difficult to assess as a comprehensive investigation establishing evaluation metrics for this type of reporting was limited. In 2016, Stogryn et al. established 75 quality indicators (QIs) for Roux-en-Y Gastric Bypass (RYGB) using a multidisciplinary, three-round national Delphi process. The QIs are separated under nine subheadings, as described previously [19].

The general reporting literature has also extensively analyzed the quality of operative notes created by both staff surgeons and surgical trainees. For example, Waubenet et al. evaluated and compared resident and attending operative reports created after watching a video of laparoscopic cholecystectomy [20]. They report that residents routinely describe more elements of the procedure than their attendings (56% vs 46%, respectively) but both groups underreport critical elements of the procedure such as operative complications. Similar reports from our study’s institution echo these results [1-2]. Again, in contrast to the general reporting literature, no investigation has assessed the completeness of learner narrative dictations in bariatric surgery and compared them to that of staff bariatric surgeons.

The purpose of this investigation was to analyze and compare the completeness of bariatric fellow and staff surgeon Roux-en-Y Gastric Bypass narrative dictations against nationally derived, validated, and reliable quality indicators for this procedure.

The results of this investigation have been previously presented in a poster (Stogryn S, Hardy K, Vergis A. Fellow and Attending Surgeon Operative Notes are Deficient in Reporting Established Quality Indicators for Roux-en-Y Gastric Bypass. American Society of Metabolic and Bariatric Surgery Obesity week, New Orleans, November 2016).

## Materials and methods

A total of 21 bariatric fellow reports and 21 attending RYGB narrative reports were analyzed against checklist QIs. These checklist QIs have been previously validated and have a high inter-rater agreement at the study institution, as shown in Table 1 [18-19].

**Table 1:** Quality indicators for RYGB RYGB – Roux-en-Y Gastric Bypass, BMI – Body Mass Index, GI - Gastrointestinal, EBL – Estimated Blood Loss, DVT – Deep Vein Thrombosis

Headings	# Items	Quality Indicators
Demographics	12 items	Procedure date, Report date, Reported by, Pre/postoperative dx, Procedure planned/performed, Mesenteric defects closed, Attending surgeon, First assistant, Second assistant, Anesthesia
Patient Details	8 items	Patient Name, Patient Age/Sex, Height (cm), Pre-operative weight (kg), Pre-operative BMI (kg/m2), Comorbidities, Additional GI conditions, Previous abdominal surgeries
Pre-operative Events	5 items	Preoperative diet type, Preoperative diet duration, Weight loss on diet (kg), Weight post diet (kg), Preoperative endoscopy
Operative Details	13 items	Additional procedures performed, Preoperative antibiotics, Thromboprophylaxis, Sequential compression devices, Compress stockings, Skin preparation type, Time-out performed, Patient position, Pneumoperitoneum, Pneumoperitoneum complication, Final port placement, Laparoscopy findings, Omental division
Small Bowel Division Details	3 items	Small bowel division stapler, Biliary length (cm), Roux length (cm)
Entero-enterostomy Details	7 items	Number of entero-enterostomy staplers type, Entero-enterostomy, Staplers closure technique, Closure suture/method, Closure staplers, Anti-obstruction stitch, Mesenteric closure
Gastrojejunostomy Details	14 items	Gastrojejunostomy position, Length of pouch (cm), Bougie type/size, Number of gastric pouch staplers, Type of gastric pouch staplers, Use of clips on pouch, Anastomotic technique, Anastomotic staplers, Closure sutures/method, Hand-sewn anastomotic sutures, Closure staplers, Leak test, Gastroscopy Peterson’s space closure
Closure Details	10 items	Port/skin closure, Skin closure, Intraoperative complications, Location complication, Unexpected findings/events, Sponge/instrument count, Drains placed, EBL (cc), Operative time (h:min), Pathological/microbiology specimen
Post-operative Details	3 items	Postoperative condition, Postoperative DVT prophylaxis, Additional information/notes (free text)

Quality assessment

A retrospective assessment of local narrative operative reports dictated by staff surgeons and bariatric surgical fellows was performed. These reports were selected at random from RYGB performed by surgeons and fellows over the time frame between 2013 and 2016 at the Manitoba Centre for Metabolic and Bariatric Surgery in Winnipeg, Manitoba. This publically funded bariatric surgery program was established in 2010, employs four bariatric surgeons, and currently performs approximately 210 RYGB per year. The program is the main teaching center for the University of Manitoba Minimally Invasive and Bariatric Surgery fellowship program. This program takes one fellow per year. All fellows are board certified and had not been in independent practice prior to starting their fellowship. This time frame was selected to reflect a well-established bariatric program and not be confounded by learning curve or significant practice adjustments over time and to ensure that reports from multiple trainees were assessed.

The quality of the narrative reports was evaluated against checklist QIs. The list consists of 75 individual items in a checklist format under nine subheadings. These indicators include demographic, preoperative, intraoperative, and postoperative items that were determined by a multidisciplinary group to be important to include in an RYGB operative report. Items were marked as “1” for present, “0” for absent, and “N/A” for not applicable elements. The total present items were tallied and a percent completeness score was calculated. “Not applicable” elements were excluded from the total. Subsection analyses were additionally performed to identify areas of strength and weakness. Data extractors were blinded to the author of the report and whether it was dictated by a fellow or staff surgeon.

Ethics

University of Manitoba institutional research ethics approval was obtained prior to the commencement of this study.

## Results

A total of 21 fellow and 21 attending reports were evaluated. This included dictations from four fellows and four staff surgeons. Each individual was equally represented.

Fellows had a mean completion of 66.4% +/- 3.1% as compared to 61.5% +/- 7.6% for attendings (p<0.0001). Fellows statistically outperformed attendings on all subsections except patient, closure, and postoperative details. Attendings statistically outperformed fellows on closure details only (63.8 +/- 7.5 vs 50.5 +/- 12.0, p=0.002).

Both fellows and attendings' most complete subsections were the team demographics, small bowel division, and entero-enterostomy details. Both groups shared patient details, preoperative events, and postoperative details as their poorest performing subsections. A summary of the quality analysis is listed in Table 2.

**Table 2:** Completeness of fellow vs attending operative reports

Subsection	#Items	Fellow completion (mean% +/- SD)	Attending completion (mean% +/- SD)	p value
Overall	75	66.4 +/- 3.1	61.5 +/- 7.6	<0.001
Team Demographics	12	85.3 +/- 3.6	69.4 +/- 14.8	<0.001
Patient Details	8	34.5 +/- 8.75	40.5 +/- 10.4	0.464
Preoperative Events	5	44.8 +/- 12.5	28.6 +/- 22.4	0.002
Operative Details	13	69.6 +/- 5.1	60.5 +/- 19.2	<0.001
Small Bowel Division Details	3	100+/- 0.0	98.3 +/- 7.3	0.042
Entero-enterostomy Details	7	85.7 +/- 0.0	72.1 +/- 3.1	0.042
Gastrojejunostomy Details	14	71.8 +/- 4.2	69.1 +/- 6.9	0.007
Closure Details	10	50.5 +/- 12.0	63.8 +/- 7.5	0.002
Postoperative Details	3	47.7 +/- 16.9	39.7 +/- 25.0	0.163

## Discussion

This investigation assessed the completeness of bariatric surgery fellow and attending operative reports. Trainees statistically outperformed their attendings overall and in all subsections, except for patient, postoperative, and closure details. The clinical significance of the absolute difference in overall scores is unknown but it is likely modest.

However, there were several subsections where there was a higher disparity in scores between trainee and staff, respectively. These include (%): "demographics" (85.3 vs 69.4), preoperative events (44.8 vs 28.6), operative details (69.6 vs 60.5), entero-enterostomy details (85.7 vs 72.1), and closure details (50.5 vs 63.8). One could speculate that the differences seen in "demographics" and preoperative events are due to the fellow focusing on the more immediate aspects of the case at hand (e.g. that they were the assistant) and that they are expected to review the clinic chart immediately prior to the operation, as they are likely meeting the patient for the first time. As such, this information is more readily at hand for the fellow as compared to the staff person who will less likely review, for example, the patients' preoperative diet immediately before the operation. The differences in operative and entero-enterostomy details may be related to the reporters’ familiarity with the procedures. For example, a novice may be more likely to record the number of stapler firings used because they are unsure of its significance in an attempt to be over rather than underinclusive. Whereas the average staff person may personally not feel the absolute number is relevant, even though this is contrary to consensus-derived opinion. In contrast, closure details were more completely reported for attendings than for fellows. This may be because they include several items that may be relevant in the outpatient postoperative setting (whether a drain needs to be removed or if there is pathology to review) or in case of an audit or medico-legal concerns (sponge/instrument counts). This may be less relevant to the trainee who is not necessarily charged with follow-up of the patient.

The trend demonstrated in this investigation is similar to Edhemovic’s, which showed trainees notes were more complete but contrasts Novitsky’s, which showed attendings were more complete and accurate [3,21]. What is more relevant though, is that both groups did not report greater than the one-third of items deemed necessary for inclusion in the RYGB operative by a national panel of expert bariatric surgeons [18]. This is significant because quality improvement initiatives in narrative reporting continue to prove largely unsuccessful in spite of more than 20 years of investigation [22].

Available data suggest that quality improvement processes aimed at surgeons and physicians only result in temporary improvements in clinical notes. This is likely because processes are not implemented into the surgical culture despite the acknowledged importance of this form of documentation [9-11,18]. The education that surgeons receive in operative notation is also a critical aspect for discussion. The literature review has shown that only a small proportion of surgical programs offer formal instruction in operative reporting despite the demonstrated need for improvement in trainee operative documentation skills [17]. The methods trainees use to teach themselves dictation skills are also questionable. Survey data indicate that not only do the majority of trainees believe their dictations need improvement and they rarely receive feedback on them, but that 80% learn to dictate by reading old reports done by their fellow residents and staff [16]. Learning by emulation relies on the assumption that those senior to you are reporting adequately. This assumption is discordant with the results of this study and the prevailing literature. This can only serve to perpetuate the low quality of operative dictation. This is particularly concerning because communication is deemed a core competency by the Accreditation Council of Graduate Medicine, The Royal College of Physicians and Surgeons of Canada, and most other accrediting bodies. Clearly, the operative note is a fundamental form of communication for the surgeon.

Specific to bariatric surgery, narrative reporting for RYGB has followed similar trends when compared to the general reporting literature. This investigation has shown that both trainees and staff surgeons incompletely include items deemed necessary when recording the structure and processes for patients undergoing the care pathways in bariatric surgical programs. These findings are consistent with previous results suggesting that NRs are of poor quality in bariatric surgery and with a national survey of bariatric surgeons who express concern that bariatric operative reports are of unacceptable quality and could potentially lead to poor patient care [15,18,23].

Importantly, the quality indicators used herein were specifically designed as benchmark criteria for the RYGB operative report itself [18]. This is in contrast to prior investigation [15,24]. The reader may argue that the quality indicators assessed may be available elsewhere in the medical records, such as nursing or clinic notes, and, as such, sections where surgeons and trainees performed poorly (e.g., preoperative events) are less important. However, it must be remembered that experts in this field have deemed it necessary to record the pre, intra, and postoperative course of these patients in a single, concise, and readily available document. This is especially critical in times of complications, particularly if the patient is presenting from another institution or country [19].

There have been several investigations in the general operative reporting literature that may be applicable in improving operative reporting for RYGB in bariatric programs. In their review, Dumitra et al. identified four educational interventions in the literature aimed at improving operative notes [17]. These have ranged from focused didactic sessions and laminated pocket template cards to computerized synoptic reporting [2,7,25-26]. Not surprisingly, the educational interventions improved performance. Unfortunately, the didactic sessions' effect on performance is not known in the long term. It is likely that the results of the templates and computerized synoptic reporting would be stable if their use was continued.

The use of computerized synoptic reporting has received considerable attention in the reporting literature. An early computerized synoptic reporting system called WebSMR was able to increase data element reporting for rectal cancer surgery from 46% to 99% [3]. These findings have been replicated across many procedures, including RYGB, as demonstrated at this institution [5,9-11,27]. From an educational standpoint, there is a concern that using synoptic reporting robs the learner of an important cognitive task analysis tool. That is, the act of recalling, organizing, and then explaining the operative procedure may, in itself, be a valuable tool that aids the learner in understanding and integrating the reasons for, and performance of, the procedure. It also affords an opportunity for feedback to be provided, not only for the learner’s skills in dictation but also for clarifying the salient points of the procedure.

The major limitation of this preliminary investigation is that it is drawn from a single center with only four staff surgeons and fellows and it may not be representative of the broader surgical communities practice. The next steps would be to analyze reports from multiple centers with a broader pool of trainees and surgeons.

We suspect, however, that the results would be similar and would follow similar trends, as borne out in the literature. Further, we suspect that education for RYGB operative reporting is deficient and needs to be improved if traditional narrative reporting is to continue in practice. Alternatively, it may be time for surgeons and educators to accept that the appetite to tangibly improve narrative operative dictation does not exist and that alternative communication forms, such as synoptic reporting, must be more widely implemented.

## Conclusions

Bariatric surgery trainees statistically outperform their attending surgeons in completion rates for RYGB operative dictations in this preliminary study. The clinical significance of this between the two groups is unknown. However, both groups are deficient in reporting at least one-third of the items deemed essential to an RYGB operative report by content experts in bariatric surgery. This suggests a need for further study and likely education in RYGB operative dictation for both practicing surgeons and trainees. It also lends interest in exploring alternative forms of operative communication, such as synoptic operative reporting, in bariatric surgery.
